# Climate change and health in urban informal settlements in low- and middle-income countries – a scoping review of health impacts and adaptation strategies

**DOI:** 10.1080/16549716.2021.1908064

**Published:** 2021-04-13

**Authors:** Frederikke Højgaard Borg, Johanne Greibe Andersen, Catherine Karekezi, Gerald Yonga, Peter Furu, Per Kallestrup, Christian Kraef

**Affiliations:** a Centre for Global Health, Department of Public Health, Aarhus University, Aarhus, Denmark; b Danish Non-communicable Diseases Alliance, Copenhagen, Denmark; c Kenya Diabetes Management and Information Centre, Nairobi, Kenya; d Non-communicable Diseases Alliance Kenya, Nairobi, Kenya; e Medical Faculty, University of Nairobi, Nairobi, Kenya; f Global Health Section, Department of Public Health, University of Copenhagen, Copenhagen, Denmark; g Heidelberg Institute of Global Health, University of Heidelberg, Germany

**Keywords:** Climate change, informal settlements, non-communicable diseases, communicable diseases, low- and middle-income countries

## Abstract

**Background**: Climate change affects human health with those with the least resources being most vulnerable. However, little is known about the impact of climate change on human health and effective adaptation methods in informal settlements in low- and middle-income countries.

**Objective**: The objective of this scoping review was to identify, characterize, and summarize research evidence on the impact of climate change on human health in informal settlements and the available adaptation methods and interventions.

**Method**: A scoping review was conducted using the Arksey and O’Malley framework. The four bibliographic databases PubMed, Web of Science, Embase, and the Cochrane library were searched. Eligibility criteria were all types of peer-reviewed publications reporting on climate change or related extreme weather events (as defined by the United Nations Framework Convention on Climate Change), informal settlements (as defined by UN-Habitat), low- and middle-income countries (as defined by the World Bank) and immediate human health impacts. Review selection and characterization were performed by two independent reviewers using a predefined form.

**Results**: Out of 1197 studies initially identified, 15 articles were retained. We found nine original research articles, and six reviews, commentaries, and editorials. The articles were reporting on the exposures flooding, temperature changes and perceptions of climate change with health outcomes broadly categorized as mental health, communicable diseases, and non-communicable diseases. Six studies had a geographical focus on Asia, four on Africa, and one on South America, the remaining four articles had no geographical focus. One article investigated an adaptation method for heat exposure. Serval other adaptation methods were proposed, though they were not investigated by the articles in this review.

**Conclusion**: There is a paucity of original research and solid study designs. Further studies are needed to improve the understanding of the impact, the most effective adaptation methods and to inform policy making.

## Background

According to the United Nations and its Intergovernmental Panel on Climate Change (IPCC), climate change is one of the major global challenges of our time due to the unprecedented scale and worldwide impact [1]. The emission of greenhouse gasses (GHG) is substantially changing the climate of the planet and human activities are regarded as the main cause of GHG emissions [2,3]. The United Nations Framework Convention on Climate Change (UNFCCC), in its article 1, defines climate change as “A change of climate which is attributed directly or indirectly to human activity that alters the composition of the global atmosphere and which is in addition to natural climate variability observed over comparable time periods” [4].

The climate crisis is moreover a health crisis [3,5]. According to WHO, climate change affects several determinants of health, for example, air quality, availability of safe drinking water, food supplies, and secure shelter. Climate change is expected to cause approximately 250.000 additional deaths per year between 2030 and 2050 related only to malaria, diarrhea, malnutrition and heat stress [6,7]. Consequences of climate change can be of direct effect such as extreme weather events, droughts, (river) floodings, landslides, and sea level rises, in particular in light of many megacities and their slums on the direct seashore, and indirect effects (e.g. mediated by poor water quality and malnutrition due to failed and poor harvests) and cause an increase in communicable diseases (CDS) such as diarrheal disease and dengue but also mental disorders and non-communicable diseases (NCDs) such as cardiovascular and respiratory diseases [7,8].

Urban areas, including slums, attract substantial population flow from rural areas due to factors such as economic and educational opportunities and better health care [9]. Many urban areas across the world are vulnerable to climate change and the health risks it poses to their populations through effects such as sea level rises and floodings, heat islands, and the increase of vector borne diseases [10,11]. The Fifth Assessment Report of the IPCC stated that “Urbanization and climate change may work synergistically to increase disease burdens’ [12,13]. It is estimated that more than 1 billion people worldwide live in slum areas [9]. Slum households are defined by the UN-Habitat as ‘One in which the inhabitants suffer one or more of the following “household deprivations”: lack of access to an improved water source, lack of access to improved sanitation facilities, lack of sufficient living area, lack of housing durability and lack of security of tenure’ [14,p.], and informal settlements as ‘Urban neighborhoods that developed outside the formal system recording land ownership, land tenure and a range of regulations relating to planning and land use, built structures and health and safety’ [14]. Terms such as slum area, informal settlement, and low-income community, are often, but not always, used interchangeably [15,16]. In this article informal settlement and slum will be treated as synonyms, (despite different definitions by UN-habitat) since several settlements might meet both definitions [17]. Informal settlements are often placed in hazardous areas, such as landslide- or flood-prone locations or polluted environments [18]. Furthermore, climate change is promoting rural to urban migration (e.g. due to a reduction in agricultural livelihoods) and failure to adapt to the increasing low-income urban population may intensify the health inequalities related to inadequate housing, lack of infrastructure, and adequate sanitation [19]. An important challenge for climate change adaptation efforts is to build health resilience for the inhabitants of informal settlements as they are particularly vulnerable to the health effects of climate change [14]. However, access to health care can often be better than in rural areas where many migrants originate [20].

The inhabitants of informal settlements often have low access to healthcare compared to more affluent urban areas, which is compounded by various health problems driven by lack of sanitation, overcrowding, and the general harsh physical and social living conditions of the informal settlements [21]. Prevalent health conditions among residents in many informal settlements are diarrhea, HIV, tuberculosis, pneumonia, malaria, and mental health problems [18,21,22]. Poor health conditions and housing make inhabitants of informal settlements especially vulnerable to the health effects of climate change [23]. Moreover, the residents of informal settlements are a population often neglected by research, resulting in very limited availability of health-related data and information [24,25]. Currently our understanding of the health impact of climate change and the available adaptation methods is insufficient to facilitate an adequate public health response. Thus, it is important to identify the nature of the available evidence on the health impact of climate change in informal settlements and the available adaptation methods. To the best of our knowledge no scoping or systematic review of the available evidence has been conducted before.

### Research questions and objectives

*The objectives of this study are*:

- To describe the existing peer-reviewed literature on climate change and health in urban informal settlements.
- To review and summarize the available evidence on the health impacts of climate change in urban informal settlements in low- and middle-income countries (LMIC).
- To review and summarize the available scientific evidence on climate change adaptation in urban informal settlements in LMICs.

Specific research questions are:

- What is published according to publication type, methods, and data sources?
- What is the geographical focus of the published literature?
- What kind of climate change exposures have been investigated in relation to health in urban informal settlements?
- What health impacts have been described in relation to climate change for the residents of informal settlements?
- What health-related adaptation methods are described?
- What recommendations for research and policy are made?

## Methods

A scoping review is a method of reviewing evidence-based research to identify knowledge gaps, scope a body of literature, clarify concepts or to investigate research conduct [26]. We chose the scoping review method (over a more focused systematic review) to allow creating an overview of a potentially large and diverse body of literature pertaining to this broad topic, and to identify research gaps. The scoping review framework developed by Arksey and O’Malley in 2005 and updated by Peters et al. in 2015 was applied for this study [27,28]. A research protocol was created *a-priori* and consecutively followed in conducting this scoping review. The PRISMA extension for scoping reviews (PRISMA-ScR) checklist was used in developing the protocol and writing the manuscript [29].

### Databases and search strategy

The databases PubMed, Web of Science, Embase, and the Cochrane library were searched. In addition, we screened the reference list of the health-related chapter 11 of working group 2 of the IPCC fifth Assessment Report. Reference lists of included publications were furthermore searched for relevant articles. The search strategy was based on the three main areas of interest: climate change, informal settlements, and health. Climate change was defined according to the definition by UNFCCC [4] including extreme weather events defined by IPCC [30], slums and informal settlements were defined according to UN-Habitat’s definitions while health was defined as immediate human health outcomes [14,31]. The search strategy for PubMed (Table 1) was based on free text and Medical Subject Headings (MeSH) terms. Search strategy for Web of Science presented in table 2.

**Table 1.** Search terms used in PubMed, Embase and Cochrane

Category	PubMed, Embase and Cochrane Search Strategy
Climate Change	(‘climate change’ OR ‘climate variabil*’ OR ‘global warming’ OR ‘greenhouse effect*’ OR ‘greenhouse gas emission*’ OR GHGE OR ‘heat wave*’ OR heatwave* OR ‘high temperature*’ OR drought* OR flood* OR ‘climate induced’ OR ‘climate related disaster*’ OR ‘storm*’ OR ‘typhoo*’ OR ‘hurricane*’ OR ‘cyclone*’ OR ‘sea level rise*’)
Informal Settlements	(slum* OR settlement*)
Health	(health* OR well-being OR wellbeing OR prevalence OR incidence OR risk OR rate OR mortality OR morbidity OR obesity OR malnutrition OR malnour* OR overweight OR over-weight OR underweight OR under-weight OR nutrient OR iron OR iodine OR ‘vitamin d’ OR ‘vitamin b12’ OR calcium OR ‘vitamin a’ OR zinc OR magnesium OR deficien* OR shortage* OR value* OR anemia OR anaemia OR hypertension OR ‘blood pressure’ OR BP OR stroke OR diabetes OR ICH OR ‘heart disease’ OR CKD OR ‘kidney disease’ OR chronic OR cardiovascular OR cardio-vascular OR malaria OR dengue OR vector OR cholera OR cancer)

### Criteria for inclusion and exclusion

The following inclusion criteria were used in selecting the studies:

- Low- and middle-income countries (including both lower- and upper-middle-income countries as defined by the World Bank [32])
- Urban informal settlements or urban slums (defined by UN-Habitat [14,31])
- Human health impacts or adaptation strategies to protect human health
- Climate change as a central element in the publication (the authors must link their investigations to climate change, or an extreme weather event linked to climate change)

Only peer-reviewed articles (including original quantitative and qualitative studies, systematic reviews, editorials, and commentaries) published between 1 January 1990 and 6^th^ of July 2020 in English, French, German, and Danish were included.

The following exclusion criteria were used:

- Book chapters and grey literature (dissertations, conference proceedings, reports etc.)
- Publications with primary focus on the environment without human health outcomes

### Selection process

First all duplicates were removed. Then, two reviewers independently screened titles and abstracts of the identified papers for relevance. Those not meeting the inclusion criteria were excluded. Full texts were obtained for all studies appearing to meet the inclusion criteria and a final selection was made after evaluation of the full article. Any disagreements or doubts between the two reviewers were discussed and evaluated before a final decision was made.

### Data collection and extraction

Search results were merged, and duplicates were removed using citation software Zotero. Data about author, first and last author affiliation, year of publication, geographical focus of the publication, publication type, type of health effect studied, climate exposure studied, population, method, and data sources used, outcome measures, interventions, and recommendations (if described) were collected and transferred into an Excel file and coded to facilitate analysis and the development of summarizing tables and infographics.

Articles were sorted and presented in two categories, original research, and other literature (reviews, commentaries, and editorials). In the results section under ‘How are the informal settlements impacted?’, focus was on the original research papers, as they provided investigations of specific climate exposures and health effects. Reviews, commentaries, and editorials are analyzed later in the manuscript.

### Ethics

No ethical approval was required as only secondary data was investigated and used.

## Results

### Search results

**Figure 1.**
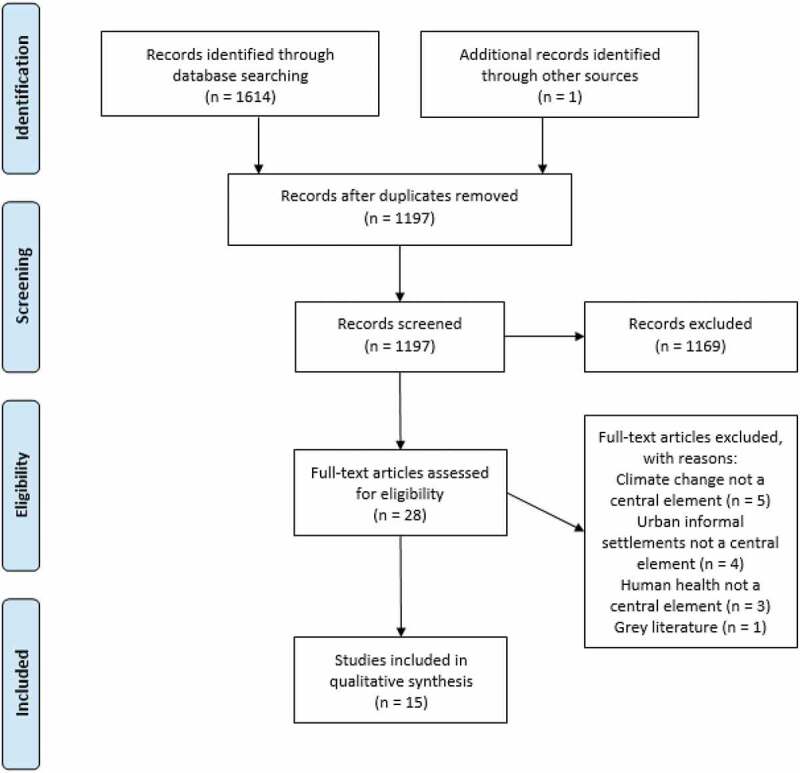
PRISMA 2009 flow diagram of the literature selection process. Moher D, Liberati A, Tetzlaff J, Altman DG, The PRISMA Group (2009). Preferred reporting items for systematic reviews and meta-analyses: The PRISMA statement. PLoS Med 6(7): e10000

The electronic searches yielded 1,197 articles after duplicates were removed (Figure 1). Numerous articles on marine biology were identified by the search strategy furthermore many articles investigated climate change and health in high-income countries; therefore, 1,169 citations were excluded during the screening of titles and abstracts. Full texts were retrieved for 28 studies. Of these, 13 studies were excluded during assessment of the full text. 15 articles were eligible for inclusion in this review [13,23,33–45].

### Characteristics of included studies

**Figure 2.**
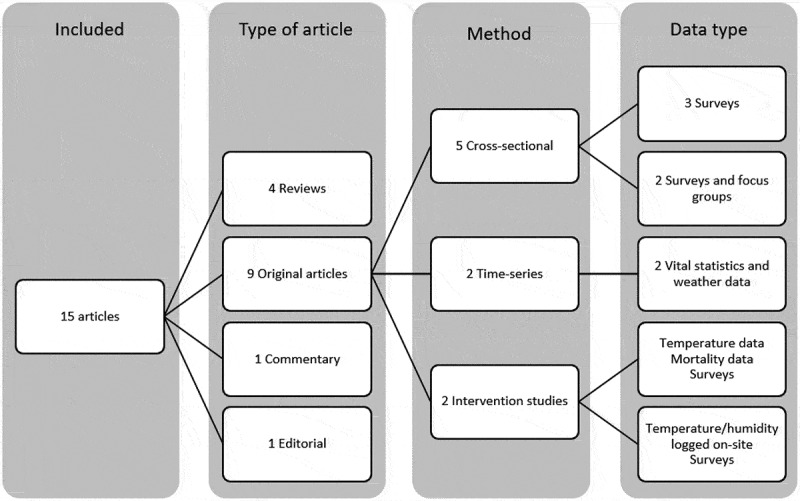
Provides an overview of the publication types, methods, and data sources used in the studies

All articles except one [13] were published between 2010 and 2020. Nine original research articles, four reviews, and two commentaries or editorials were identified. Seven studies used observational study design (five cross-sectional, two used time-series models), one used interventional method, and one described the process for developing an intervention. Four of the nine original research papers used surveys combined with other data types, three used surveys as the only data source (Figure 2).

**Figure 3.**
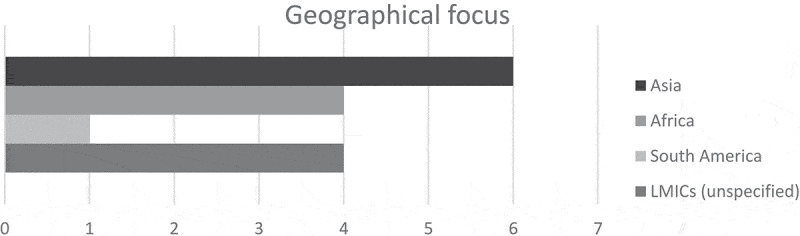
The distribution of geographical focus of the identified papers

The first authors were primarily from high-income countries (n = 9/15), although six

were based in LMICs. The last authors were in all except three cases from high-income countries. Most studies focused on cities in Asia; however, both Africa and South America were represented among the included articles. Four articles had no specific geographical focus (Figure 3). A summary overview of all the included articles is presented in Table S1.

### How are the informal settlements impacted?

The included original articles described impacts from various climate exposures (temperature related, flooding etc.) and investigated a range of health outcomes in informal settlements (mortality, communicable diseases, mental disorders etc.). In this section the populations, climate exposures, health outcomes and primary findings by the original articles included will be described. Table 3 provides an overview of the original research articles and their characteristics.

**Table 2.** Search terms used in web of science

Category	Web of Science Search Strategy
Climate Change	(‘climate change’ OR ‘climate variabil*’ OR ‘global warming’ OR ‘greenhouse effect*’ OR ‘heat wave*’ OR heatwave* OR drought* OR flood* OR ‘climate induced’ OR ‘climate related disaster*’ OR ‘storm*’ OR ‘typhoo*’ OR ‘hurricane*’ OR ‘cyclone*’ OR ‘sea level rise*’)
Informal Settlements	(slum* OR settlement*)
Health	(mortality OR morbidity OR obesity OR malnutrition OR malnour* OR overweight OR over-weight OR underweight OR under-weight OR nutrient OR iron OR iodine OR ‘vitamin d’ OR ‘vitamin b12’ OR calcium OR ‘vitamin a’ OR zinc OR magnesium OR anemia OR anaemia OR hypertension OR ‘blood pressure’ OR stroke OR diabetes OR ICH OR ‘heart disease’ OR CKD OR ‘kidney disease’ OR chronic OR cardiovascular OR cardio-vascular OR malaria OR dengue OR vector OR cholera OR cancer)

**Table 3.** Characteristics of the original articles

Author (year)	Study site	Climate exposure	Health effect	Outcome measures	Adaptations	Recommendations
**Original research articles**						
Bambrick H. *et al*. (2015)	Shashemene (Ethiopia)	Climate change(not specified)	Indirect effects: Food insecurity and malnutrition CDS: Changes to infectious disease transmission e.g. diarrhea, typhoid, and malaria	Identification of climate-sensitive health conditions and the local hazards that may affect the informal urban communities	* Improved sanitation and water supply * Vaccination	Research: Local research to inform national policy Research taking community needs into consideration.
Contreras C. *et al*. (2018)	Lima (Peru)	Flooding	Indirect effects: Mental Health: depression and domestic violence	Prevalence of depression and domestic violence. Persons accepting the accompaniment to government services	Elements of the intervention (1) screening for depression and domestic violence, (2) children’s activities to strengthen social and emotional skills and diminish stress, (3) participatory theater activities to support conflict resolution and community resilience, and (4) community health worker (CHW) accompaniment to government health services.	N/A
Egondi T. *et al*. (2015)	Nairobi (Kenya)	Temperature variation, heat waves, and cold spells	Direct effect: Years of Life Lost	Association of temperature and Years of Life Lost	* Improvement of housing conditions and building standards * Create awareness of the dangers of extreme temperatures	Research: Individual level studies to establish vulnerable groups and help in designing adaptive strategies.
Egondi T. *et al*. (2012)	Nairobi (Kenya)	Temperature and rainfall	Direct effects: Mortality	Association of temperature, rainfall, and mortality	* Proper housing and clothing	Research: Epidemiologic studies that incorporate archived climatological and environmental data in modeling specific health outcomes in vulnerable populations
Khan M. *et al*. (2014)	Dhaka(Bangladesh)	Flooding	Indirect effects: Poor mental well-being, malnutrition CDS: diarrhea and gastric disease NCD: High blood pressure, diabetes, heart disease	Affected by flood/stagnant water. Health problems. Urban/rural impact	* Hand washing with soap, proper disposal of fecal material, and the use of modern toilets	Policy: Availability of essential equipment e.g. power generators and water pumps Allocation of alternative less vulnerable settlement areas
Knowlton K. *et al*. (2014)	Ahmedabad (India)	Heat waves	N/A	N/A	* Community outreach to build public awareness* Early warning systems and weather forecasting *Capacity building among health care professionals	Policy: A seven-phase model described in the article can be used as a template for developing other adaptation projects for extreme weather events in lower-resource settings
Toan D. *et al*. (2014)	Hanoi (Vietnam)	Climate change (not specified)	Illness (not specified) CDS: Emerging diseases (dengue, Japanese encephalitis)	Perceptions and knowledge of climate change and health. Perceived illness among family members	N/A	Policy: Make use of community groups as climate change communication channels Research: Studies where perceptions are checked against real health data could provide more precision
Tran K. *et al*. (2013)	Ahmedabad (India)	Extreme heat	Direct effects: Heat-related symptoms, Heat-related illness	Self-reported heat-related illness and heat-related symptoms at the individual level	* Inform people about the dangers of extreme heat * Capacity building among health care professionals and CHW	Research: Future research should assess exposure-outcome associations and focus on intervention implementation and evaluation
Vellingiri S. *et al*. (2020)	Ahmedabad (India)	Temperature	Direct effect: Heat-related illness	Temperatures in intervention and nonintervention homes	Selected cool roof technologies can reduce indoor temperatures	Research: Future studies with larger sample sizes and better research designs
**Reviews, Commentaries, View-Points, and Editorials**						
Corburn J. *et al*. (2017)	N/A	Climate change (not specified)	Health (not specified)	Health benefits of slum upgrading	Improved flood control by upgrading of infrastructure and housing	Policy: Having a supportive state policy framework can enhance long-term impacts from slum upgrading upon population health
Munslow B. *et al*. (2010)	Asia	Climate change (various)	Health (not specified)	N/A	* Early warning systems * Disaster preparedness	Policy: Creation of multi stakeholder partnerships
Scovronick N. *et al*. (2015)	N/A	Climate change (various)	Health (various)	N/A	* Land access and provision of infrastructure * Ecosystem protection	Research: The article provides an overview of study designs that have been used for research in informal settlements and only require moderate data inputs Lack of data might hinder future research
Sverdlik A. (2011)	N/A	Climate change (various)	Health (various)	N/A	* Improvement of housing conditions and building standards	Policy: Low-income groups may require special assistanceResearch: Evaluate the pathways between climate change and NCDs
**Commentaries and editorials**						
Patrick R. *et al*. (2016)	N/A	Climate change (various)	Health (various)	N/A	* Urban food production to reduce heat, food insecurity and GHE	Policy: Promoting citizen participation in decisions about the future of the global food system
Ramin B. (2009)	Africa	Climate change (various)	Health (various)	N/A	N/A	Policy: taking into account climate change and urbanization when planning public health interventionsResearch: research focused on climate-change related health outcomes among slum dwellers

*Suggested/proposed adaptation strategy, not investigated in the article

#### Populations

The size of the populations varied considerably between the studies. The smallest investigated 16 households [45] and the largest investigated entire slum areas with a population of approximately 66,000 [36]. Several of the studies were conducted on a household level, often with one individual answering for the entire household. Not all articles provided population characteristics (available population information and characteristics are presented in Table S2). In those with population characteristics available, most respondents were women [33,34,38,43], one study [44] preferred women respondents because of assumed better knowledge of the household’s health conditions, and another [45] chose to only ask the head female of the household.

#### Climate change exposures

More than half of the original research publications (n = 5/9) investigated temperature-related exposures. Three focused on temperature variations in general [36,37,45] and two focused on extreme heat events and heat waves [39,44]. Furthermore, two studies focused on flooding [34,38], and two considered perceptions of climate change e.g. which climate change exposures the inhabitants of informal settlements experience, and what exposures they perceive as risks for the future [33,43].

#### Health outcomes

Four articles studied direct health effects such as heat-related symptoms and illness [44,45] or mortality [36,37]. Indirect effects were studied by three papers, two assessed mental health [34] and wellbeing [38], and two assessed malnutrition [33,38]. Different communicable diseases were studied by three articles. One considered diarrhea, typhoid, and malaria [33], another diarrhea and gastric disease [38], and the last emerging infectious diseases namely dengue and Japanese encephalitis [43]. NCDs were considered by two studies [37,38]. One article assessed a policy planning process-oriented article and did not report health outcomes [39].

#### Primary findings by the original articles

The primary findings by the original articles will be presented aligned by climate exposure. First results related to temperature and weather variation, followed by results related to heat waves will be presented. Then results related to flooding, and finally results related to perceptions of climate change.

In Nairobi, a J-shaped curve of association between Years of Life Lost (YLL) and lower temperatures was found. Colder temperatures appeared more harmful [36]. Another study in Nairobi similarly found a J-shaped curve for temperature and mortality associations, with the elderly especially vulnerable to cold temperatures. Children under the age of 5 and people with NCDs were here found more vulnerable to higher temperatures compared to the rest of the population. Furthermore, it was found that mortality increased with rainfall in a linear model [37].

A study in Ahmedabad found that heat-related symptoms (small blisters or pimples, dry mouth, fatigue, leg cramps, heavy sweating, intense thirst, rapid heartbeat, headache, and leg swelling) were reported by one-fifth of the respondents. Heat-related illnesses (heat rash, edema, and exhaustion, hyperthermia and heat stroke diagnosed by a healthcare provider) were reported by approximately 12%. People over the age of 60, people with medical conditions and outdoor workers, were especially vulnerable to heat-related symptoms [44]. Another study from Ahmedabad found that 45% reported heat-related illness during the summer. They investigated a cool roof intervention where standard roof types tin roof, asbestos/cement sheets, and concrete were changed with the intervention roof types to evaluate the effect on indoor temperatures. The intervention roof types were solar reflective white paint on tin, modular roofing system (Modroof), Airlite ventilation sheet with a passage to allow airflow into the house, and Thermocol insulation (a false secondary ceiling). It was found that Thermocol insulation, solar reflective white paint, and Modroof reduced indoor temperatures [45]. Additionally, one study from Ahmedabad was process oriented and described the development of a heat action plan; however, the plan was not evaluated in the study [39].

In Lima an intervention study evaluated depression and domestic violence in relation to a flooding episode and found depression in 10% and reported domestic violence in 36% of the screened participants. A model for a small-scale response to disasters was created to identify and link individuals with mental health problems to health services. 50% accepted help after screening for depression and domestic violence, and accompaniment to government health services [34]. In Dhaka, it was found that people living in areas affected by flooding and stagnant water (FSW) reported poor mental wellbeing, more communicable diseases (e.g. diarrhea), and more health symptoms in general compared to people not affected by FSW. Health symptoms included fever, weakness, and cold/cough. Furthermore, informal settlements were more affected by FSW than rural areas [38].

The population’s perception of climate change and health was studied in Hanoi. They found that one third of respondents reported increased illness among family members compared to earlier. Additionally, they reported a perceived increase in emerging infectious diseases such as dengue and Japanese encephalitis among the respondents [43]. The same was done in Ethiopia. They found that the residents of informal settlements perceived increased food insecurity and increased infectious disease transmission (e.g. malaria, typhoid, and diarrhea) were the most likely threats to their health affected by climate change [33].

### Which intervention or adaptation methods were described?

Two of the original papers assessed interventions or adaptation methods [34,45], though no direct effects on health outcomes were described. Several of the other articles suggested possible adaptation methods for building health resilience in relation to climate change but they were only proposed and not investigated further.

#### Slum upgrading

Upgrading strategies were proposed as an adaptation strategy in seven articles. The effect of cooling roofs for reducing indoor temperatures was investigated in one study. Thermocol insulation, solar reflective white paint, and Modroof were shown to reduce indoor temperatures but direct health outcomes e.g. heat-related illness were only evaluated before the intervention [45]. In a review, it was described that climate change resilience can be built through slum upgrading for example improved flood control by upgrading infrastructure and housing [35]. Other slum upgrading strategies mentioned were improved water supply and sanitation [33], improvement of housing conditions and building standards [23,36,37], land access and provision of infrastructure [42] but they were not investigated further.

#### Health service related

Six articles proposed health service-related adaptation methods. Promotion of mental health care access was investigated in Peru [34]. It was found that case identification, through screening for depression and domestic violence after an extreme weather event, and community health worker (CHW) accompaniment to government health service could facilitate access to mental health care services for residents of informal settlements. Additionally, the following health service-related adaptation strategies were mentioned by other studies: immunization through vaccination [33], improved sanitation and hygiene through hand washing with soap [38], to create awareness and inform people about the dangers of e.g. extreme heat [36,39,44], capacity building among health care professionals and CHW [39,44] but they were not investigated further.

#### Other

In addition, four articles described other adaptation methods. According to one article urban food production can reduce the vulnerability to food insecurity and price increases in case of extreme weather events. Furthermore, it can provide climate change mitigation through the cooling effect of increased vegetation as well as less energy used for transport and storage of food [41]. Other examples were early warning systems e.g. through weather forecasting and alerting people with text messages, TV, and radio [39,40], disaster preparedness [40], and ecosystem protection [42] but they were not investigated further.

### Which recommendations were made in the publications?

One article described the development and implementation of a heat action plan. A seven-phase model described in the article can be used as a template for developing other adaptation projects for extreme weather events in lower-resource settings [39].

#### Policy

Seven articles provided recommendations for policy makers. The recommendations were to prioritize community strengthening through participation of local communities in decision-making [38,41] and as communication channels [43], and creation of multi stakeholder partnerships [40]. Furthermore, the availability of essential equipment e.g. power generators and water pumps [38], allocation of alternative less vulnerable settlement areas [38] and having a supportive policy framework were described as important for long-term slum upgrading success [35], and preventing future disasters. Furthermore, it was recommended that the dynamic relationship between climate change and urbanization should be considered when planning public health interventions in Africa [13] and one article stated that low-income groups may require special assistance [23].

#### Research

Nine articles provided recommendations for future research. Several stated that the current data are limited, and that more health data is needed to provide better knowledge on climate hazards and health outcomes [33,42,43]. One of the identified articles described study designs that have been used for research in informal settlements and only require moderate data inputs [42]. Additionally, it was recommended to identify vulnerable subgroups [36], to evaluate the pathways between climate change and NCDs [23], and to conduct studies with larger sample sizes and better research designs [45]. The researchers advocated for the necessity of further research on interventions and adaptation methods [13,33,36,37,44].

## Discussion

To the best of our knowledge, this is the first review to explore and map the literature on the health impact of climate change in urban informal settlements in LMICs. The broad search strategy identified only 15 studies in this scoping review although the search strategy had no geographical or health-specific limitations. This scarcity of evidence is in line with a review by a systematic assessment of publications with a climate change and health focus from 2016, which found certain health impacts, particularly malnutrition and non-communicable diseases (NCDs), being half that of other sectors, with approximately two-thirds of all published studies carried out in OECD countries [46].

South America was poorly represented in the studies identified, with only one article [34]. Among the original articles five out of nine (56%) performed their research in the only two cities, Nairobi (Kenya) and Ahmedabad (India). This highlights the need for research projects with a more diverse geographical distribution.

Five out of nine original articles focused their research on temperature-related climate impacts while none investigated the impact of drought or storms. This underlines the predominance of research concerning temperature variations and heat waves, and the necessity of studies regarding other climate exposures.

In global literature, communicable diseases, particularly dengue, and other vector-borne diseases are commonly investigated health outcomes in relation to climate change [47]. In this scoping review, direct health effects of climate change were the most studied health effects (n = 4), closely followed by indirect effects (n = 3), and communicable diseases (n = 3). NCDs have been identified as an emerging risk for inhabitants of urban informal settlements [23] and NCDs are furthermore suspected to be affected by climate change [48]. NCDs were only mentioned in two articles. Thus, further research on the effects of climate change on NCDs in informal settlements is warranted.

All 15 studies presented adaptation proposals or recommendations for policy makers or future research, but none investigated the impact of interventions or adaptations on direct health outcomes. One study investigated a roof type intervention where the outcome was indoor temperature. A direct health outcome e.g. prevalence of heat-related illness was only evaluated before but not after the intervention roof types were implemented. Further research of the proposed adaptations is essential, as well as studies where direct health outcomes e.g. prevalence of illness or symptoms are evaluated both before and after interventions. However, the small number of studies conducted over time point towards underlying barriers to conducting research on health impacts of climate change, such as the very long lag time between emissions, climate change, and health impacts. On the other hand, research and data acquisition in slums is likely easier than in rural areas.

The scoping review methodology chosen for this article enabled a broad approach to the topic, providing a holistic overview of the literature on climate change and health in urban informal settlements. However, due to the different study designs and methodologies of the articles included, no direct comparison (e.g. meta-analysis) between studies, geographical areas etc. was possible. The search strategy for this scoping review did not identify any articles that investigated the impact of climate change on health in relation to water- and sanitation-problems (WASH) [49] of informal settlements and identified only two [34,38] with a focus on mental health. A weakness of this review is that some infectious diseases (e.g. Japanese encephalitis), WASH, mental health and health care were not defined a-priori as important mediators and health outcomes in the search strategy. This may have potentially under-represented studies on health/health care adaptation and on certain climate-sensitive diseases and precluded the identification of relevant articles. Even though they were likely covered by the search strategy in general due to broad search terms (e.g. morbidity, mortality, vector) further research or a structured literature review would be welcome to shed further light into these important issues. Due to the nature of this scoping review no quality or bias assessment of the studies was conducted.

There was a shortage of studies based on robust health data (e.g. hospital records, surveillance data and vital statistics) with only three original articles based on these data. Several studies stated that the current lack of data (routine collected data, data from health services, completeness of data, and data about symptoms) in developing countries hinders research and understanding of the effects of climate hazards on health outcomes [33,42,43]. Therefore, one article argues for studies with modest data requirements [42]. In addition, almost all the original articles identified are observational studies. This emphasizes the need for interventional studies as recommended by many of the articles identified [13,33,36,37,44].

The populations of LMICs have much lower GHG emissions per capita than developed countries [50]. However, climate change exacerbates existing health inequities between developed and developing countries especially for the poorest, who often live in vulnerable informal settlements [50–52]. Although the increasing scientific interest in health and climate change has led to more than 2,000 scientific articles to date [47], most of these focus on climate and health in high-income countries, similar to what is seen for global funding of health research known as the ‘10/90’-gap [47,53]. Thus, the most notable research gap identified was the shortage of literature concerned with climate change and health in urban informal settlements in LMICs compared to the overall research output on the issue.

## Conclusion

The health of inhabitants of informal settlements is disproportionally affected by climate change. There is, however, a huge gap in the interest of the scientific community in studies on the impact on climate change and potential adaptive responses in informal urban settlements. This stands in starks contrast to the high vulnerability of informal urban settlements to climate change and the long list of known risk factors (e.g. inadequate access to healthcare, lack of sanitation, and overcrowding) its populations face. Most articles we identified consisted of non-evidence-based papers such as commentaries. This lack of evidence has also been pointed out by several authors emphasizing the need for more data on climate hazards and their health outcomes in informal settlements as well as studies evaluating adaptation methods and interventions to reduce the risk.

## Supplementary Material

Supplemental Material

## Paper context

Climate change is one of the biggest global health threats in the 21^st^ century and the poorest are most vulnerable. Surprisingly little is known about the impact of climate change in informal settlements in low- and middle-income countries. Therefore, we conducted the first systematic (scoping) review to provide an overview of the available scientific literature and to identify research gaps. Our results can be used to plan further research and to inform policy making.
