# Supplementary material for: Climate change and health in urban informal settlements in low- and middle-income countries – a scoping review of health impacts and adaptation strategies
Source: Glob Health Action. 2021 Apr 13;14(1):1908064. doi: 10.1080/16549716.2021.1908064 (PMC8049459; doi:10.1080/16549716.2021.1908064)
Supplement: Supplemental Material [file ZGHA_A_1908064_SM6259.docx]

# Supplementary

*Supplementary table 1: Study details from the included articles.*

| **Author + affiliation (year)** | **Last author affiliation** | **Publication type** | **Key findings** |
| --- | --- | --- | --- |
| Bambrick H.  School of Medicine, University of Western Sydney, Australia  (2015) | Faculty of Economics, Management&Accountancy, University of Malta, Malta | Original research | Climate change will intensify existing health problems in communities. The most likely threats to health arising from climate change in these communities are from increasing food insecurity and changes to infectious disease transmission. |
| Contreras C.  Socios En Salud, Lima, Peru  (2018) | Department of Global Health and Social Medicine, Harvard Medical School, Boston, USA | Original research | The post-disaster response has shed light on the inadequacy of the health system to meet the needs of these disadvantaged populations. The high burden of mental health problems encountered in these communities suggests that community-supported interventions after disasters can provide opportunities to facilitate access to care. |
| Corburn J.  Department of City and Regional Planning & School of Public Health, University of California, Berkeley  (2017) | Department of City and Regional Planning & School of Public Health, University of California, Berkeley | Review | Urban slum upgrading is a process and set of outcomes that can positively influence multiple determinants of health and potentially reduce health inequities experienced by the urban poor. We found that a majority of slum upgrading projects rarely measured how upgrading projects affected health outcomes or social determinants. |
| Egondi T.  African Population and Health Research Center, Nairobi, Kenya  (2015) | Department of Public Health and Clinical Medicine, Epidemiology and Global Health, Umeå  University, Sweden | Original research | This study shows evidence that exposure to ambient temperature variation is associated with YLL due all causes of death in Nairobi; specifically, cold temperatures appear more harmful. The findings also point to the need to think of environmental exposures in an effort to reduce disease burden among the urban poor population. |
| Egondi T.  African Population and Health Research Center, Nairobi, Kenya  (2012) | Department of Public Health and  Clinical Medicine, Epidemiology and global Health, Umea University, Sweden | Original research | Increase in temperature above the 75th percentile showed statistically significant but moderate increases in mortality in children/infants and for NCDs. The results also show strong statistically significant positive relationships between rainfall and mortality, with cumulative lagged effect over 30 days in the following groups: all ages, NCDs, and pneumonia. |
| Khan M.  Department of Public Health Medicine, School of Public Health, Bielefeld University, Germany  (2014) | Department of Public Health Medicine, School of Public Health, Bielefeld University,  Germany | Original research | Slums were more affected by the FSW as compared to rural areas, which implies higher health risks in slums and underscores the necessities of more public health interventions there. The FSW-affected area is an independent risk factor for various physical and mental health problems. |
| Knowlton K.  Natural Resources Defense Council, New York, USA  (2014) | Ahmedabad Heat and Climate Study Group, Gandhinagar, Gujarat, India | Original research | The project builds an evidence base for publications and sharing information with the local and international community and may serve as a template for other such projects to help reduce heat vulnerability, especially in lower-resource setting. |
| Munslow B.  Humanitarian Studies Programme at the Liverpool School of  Tropical Medicine, Liverpool, UK  (2010) | Humanitarian Studies Programme at the Liverpool School of  Tropical Medicine, Liverpool, UK | Review | Climate change will add to the pressures of armed conflicts and natural disasters. Poor urban communities are particularly vulnerable to adverse health effects resulting from global climate change. Whatever happens to the poorest and worst affected country will have a serious impact on all its neighbors. |
| Patrick R.  IUHPE Global Working Group on Climate Change and Health,  School of Health & Social Development, Deakin University, Australia  (2016) | Health, Nature & Sustainability  Research Group, School of Health & Social Development, Deakin University,  Australia | Commentary | The greatest burden of heat-related health mortality and morbidity will be felt among urban slum dwellers, low socio-economic groups, and minority ethnic group. |
| Ramin B.  Faculty of Medicine, University of Ottawa, Canada.  (2009) | - | Editorial | African slum dwellers are particularly vulnerable to the negative health effects of rapid urbanization and global climate change. |
| Scovronick N.  Global Change  and Sustainability Research Institute, University of the Witwatersrand,  Johannesburg, South Africa  (2015) | Department of Social and Environmental Health Research, London School  of Hygiene and Tropical Medicine, London, UK | Review | Urban populations living in informal settlements are considered inherently vulnerable to the health impacts of climate change, but our understanding is insufficient to facilitate a quantification of the risks or the evaluation of responses. |
| Sverdlik A.  International Institute for Environment and Development, London, UK  (2011) | - | Review | Possible impacts include heightened vulnerabilities to disaster, altered communicable disease patterns, or water and food insecurity. Health impacts are often overlooked in sectoral climate change assessments, and additional research is needed to inform responses and promote resilience. |
| Toan D.  Department of Biostatistics and Medical Informatics, Institute of Training for Preventive Medicine and Public  Health, Hanoi Medical University, Vietnam  (2014) | The Medical  Committee Netherlands-Vietnam, Hanoi, Vietnam | Original research | According to the majority of respondents, climate change is occurring and results in an increase of temperature during the summer and decrease of temperature during the winter, compared to 5 years ago. They also had the perception that storms, floods, deep cold, and long heat waves were manifestations of climate change. People reported that they more easily became ill now than some years ago. |
| Tran K.  Department of Environmental Health, Emory University School of Public Health, Atlanta, USA  (2013) | Department of Environmental Health, Emory University School of Public Health, Atlanta, USA | Original research | Symptoms of heat illness were reported among one fifth of respondents. Age over 60 years, having preexisting medical conditions, outdoor work location, and limited access to water or information resources were found to increase the odds of heat-related symptoms and illnesses among urban slum dwellers. |
| Vellingiri S.  Gujarat Institute of Desert Ecology, Bhuj, Gujarat, India  (2020) | Mahila Housing Sewa Trust, Ahmedabad, Gujarat, India | Original research | Cool roof technologies have a wider scope as number of informal settlements are increasing across the cities in India and other developing countries. Validated cool roof technologies can be promoted as these structures are not requires legal sanctions and easily dismantled and installed in multiple places and safeguards the investment of urban poor. |

*Supplementary table 2: Available demographic characteristics from the included articles.*

| **Author (year)** | **Population** | **Age (mean)** | **Sex** | **Educational level** |
| --- | --- | --- | --- | --- |
| Bambrick H.  (2015) | Community A: 721 people | 23.6 | 53 % female | Preschool: 97 %  Primary: 72 %  Secondary: 22 %  Tertiary: 5 % |
|  | Community B: 939 people | 18.0 | 51 % female | Preschool: 45 %  Primary: 47 %  Secondary: 7 %  Tertiary: 1 % |
| Contreras C.  (2018) | 129 people | N/A | 81 % female | N/A |
| Egondi T.  (2015) | Approx. 66.000 people | N/A | N/A | N/A |
| Egondi T.  (2012) | 60.416 people | N/A | N/A | N/A |
| Khan M.  (2014) | Affected by FSW: 2166 people | 34.8 | 47.3 % female | No education: 62.4 %  Primary: 22.1 %  6+ years: 15.5 % |
|  | Not affected by FSW: 1034 people | 35.3 | 54.6 % female | No education: 45.9 %  Primary: 23.9 %  6+ years: 29.2 % |
| Knowlton K.  (2014) | The inhabitants of Ahmedabad | N/A | N/A | N/A |
| Toan D.  (2014) | In slum area: 690 people | 51.3 | 64.2 % female | Primary or less: 20.2 %  Secondary: 29.3 %  High school: 27.5 %  College/university: 23 % |
|  | In non-slum area: 754 people | 55.6 | 65.8 % female | Primary or less: 6.8 %  Secondary: 21.6 %  High school: 28.5 %  College/university: 43.1 % |
| Tran K.  (2013) | 300 households:  1650 individuals | 26.1 | 88 % female | N/A |
| Vellingiri S.  (2020) | 16 households:  12 controls, 4 interventions | N/A | 100% female | No education: 15 %  Primary: 45 %  Professional degrees: 7 % |
